# The impacts of housing conditions on physical and mental health – A mini-review informed by a rapid conversion of evidence from Alidoust and Huang (2021)

**DOI:** 10.3389/fenvh.2024.1352580

**Published:** 2024-02-20

**Authors:** Amelia Simpson, Luís Filipe, Valerio Benedetto, James Hill

**Affiliations:** 1 Division of Health Research, Faculty of Health and Medicine, Lancaster University, Lancaster, UK; 2 Applied Health Research hub, UCLan, Preston, UK; 3 Health Determinants Research Collaboration, Blackpool Council, Blackpool, UK

**Keywords:** Housing_1_, Health_2_, Well-being_3_, Evidence Summary_4_, Critical Appraisal_5_, Policy_6_

## Abstract

This article provides a summary and critical appraisal of the systematic review conducted by Alidoust et al. (1) regarding the various effects of housing on both physical and psychological well-being. We aim to discuss the review’s findings against existing published evidence to draw out policy and practical implications. Our mini-review illuminates a wide range of housing-related factors which impact on health around which we draw evidence-based policy initiatives and implications, and outline avenues for future research. This mini-review is part of the wider Rapid Conversion of Evidence Summaries (RaCES) program which aims to critically appraise systematic reviews and highlight evidence-based policy and practice implications.

## 1 Introduction

Housing has been recognized as a key social determinant of health (2–5). There is evidence that poor housing conditions, including lack of adequate heating, structural issues, damp, and mold can negatively affect health and well-being (6–8). In England, one in five houses do not meet the Decent Homes standard, and the cost for the United Kingdom National Health Service to treat those affected by poor housing is estimated at £1.4bn per year (9). Therefore, improving housing conditions and quality could positively impact residents’ health and benefit society (9).

The systematic review by Alidoust et al. (1) aimed to identify housing features that impact on health, explore their advantages and disadvantages, and assess their implications in different contexts. As part of the wider Rapid Conversion of Evidence Summaries (RaCES) programme (10), our mini-review aims to provide a concise summary of the findings in the original systematic review on the impacts of housing conditions on health and discuss potential shortcomings in the methodology. With the rising concern of housing conditions and their impact on population health (6–8), we chose to summarize Alidoust et al. (1) systematic review due to its focus on the latest research sources and broad coverage of topics related to housing such as neighborhood or context, physical building, housing market and housing policy.

## 2 Methods of the review by Alidoust and Huang (2021)

The systematic review by Alidoust et al. (1) undertook a search in two electronic databases (Google Scholar and Scopus) for peer-reviewed published papers in the years between 2010 and 2020. The search strategy combined concepts related to housing and health or well-being. The search was run by title, abstract and keywords. The review was restricted to the first 100 results of each database (as sorted by relevance and to articles written in English). Only case studies which reported upon the health impact of housing were included in the review. The review process occurred in three stages. Initially, the papers were screened in terms of their title and abstract. Subsequently, the remaining papers were checked for eligibility through full-text screening. Lastly, relevant information was extracted from the included studies, such as the discipline of the journals, publication dates, research methods used, specific locations of case studies, the population under study, and the research findings.

## 3 Results of the review by Alidoust et al. (1)

### 3.1 Neighborhood (n = 20 studies)

Within the neighborhood theme, a better neighborhood environment (linked to aspects like access to green spaces and upkeep of buildings), attractiveness, and safety had a positive association with residents’ psychological well-being. Similar positive associations were observed in studies exploring the role of social networks and neighborhood reputation, particularly concerning levels of deprivation and wealth. The impact of housing location on residents’ well-being was explored in the included studies, finding a more positive association between residents’ well-being and living in rural rather than urban areas, and mixed evidence on the effects of living close to urban amenities, which could be seen as either convenient or stressful.

### 3.2 Building (n = 40 studies)

Different factors were explored under the building theme. Poor housing conditions were found to be negatively associated with mental well-being, in particular when housing repair and housing improvement were taken into consideration. Certain housing features were linked to positive associations with residents’ health and quality of life such as: having new window and door fixtures (e.g., associated with improved sense of safety), smoke detectors (e.g., associated with lower risk of death and disability), internet access (e.g., associated with improved mental well-being), and insulation (e.g., associated with better thermal comfort and reduced risk of developing illnesses like asthma or bronchitis). Furthermore, living in houses built with brick veneer was found to be related to lower levels of temperature variations, while living in older houses was associated with having poorer insulation and air conditioning systems. The housing type, size, and dwelling appeared to matter too, as studies found that people living in detached houses, larger accommodations, or houses with low number of occupants had better perceived health and mental well-being, and life satisfaction.

### 3.3 Policy (n = 27 studies)

In the policy section, this review evaluated the health impacts of homeownership and house stability. Homeownership (compared to being a tenant) was associated with lower stress levels, better social and economic outcomes, and mental health outcomes, although these associations were weaker for people who had mortgages. The relationship between homeownership and self-esteem was found to be negative. The evidence regarding house-related subjective well-being was inconclusive, with some studies pointing to a positive effect with homeownership, while some found no association. In the case of divorce, owning a house was found to be a negative contributing factor to subjective well-being, as it would become a financial burden.

Additionally, older populations and children’s health needs were found to be lower for those living in their own house. In the case of children, living continuously in a rented property was associated with negative behaviors such as aggressiveness. House stability was associated with life satisfaction, mental well-being, and physical health. Insecure housing was found to be a factor of stress that could have long-term implications. In cases of relocation due to regeneration policies, impacts such as poorer mental health and well-being, damaged sense of community, feeling of loss of control, and general unhappiness were found. Negative mental health effects from housing mobility tend to be stronger in later childhood, which consequently is associated with anxiety and depression among mothers.

### 3.4 Market (n=8 studies)

Different financial factors linked to housing were considered within this theme. Living in less affordable housing was linked to poorer psychological and mental well-being. Some studies indicated that lower levels of income were associated with higher energy insecurity, potentially impacting on the residents’ health. However, living in areas with higher housing costs was not found to be necessarily associated with impacts on well-being. Other factors, linked to labor market conditions, seemed to play more of a significant role. The housing market seemed to be associated with mixed impacts on residents’ health, with rising houses’ prices positively affecting homeowners’ physical health but negatively impacting both physical and mental health of tenants.

## 4 Discussion

### 4.1 Critical appraisal

We critically appraised the methods and reporting of the systematic review by Alidoust and Huang (2021) by using the Joanna Briggs Institute tool (11) (Table 1). Firstly, while the choice of the databases (Google Scholar and Scopus) to identify suitable studies may reflect the generic review question, with only two databases and only first 100 results being used it is possible that important studies were missed resulting in evidence selection bias (12–13). Secondly, no details were reported on how the data extraction process was undertaken (again limiting any repeatability) nor on any formal critical appraisal of the studies included, whose quality remains uncertain. The sporadic and unsystematic reporting of statistical significance of the results also makes it difficult to weigh how each study contributes to answering the review’s question. Moreover, no formal analysis of potential publication bias was attempted. As a result of this critical appraisal, we believe the work by Alidoust et al. (1) aligns more closely with a scoping review. This determination is drawn from the methods used, for example, the review restricted databases to only two, limited the inclusion studies based on publication year, and lack of quality assessment for the included studies (14). We highlight the need for transparency in titles and reporting to avoid potential confusion and contribute to a more accurate understanding of methodologies in evidence reviews.

### 4.2 Implications for policy

We aimed to put the findings of the review by Alidoust et al. (1) in context to current policy and practice, as outlined below according to the specific housing-related theme.

#### 4.2.1 Neighborhood

One housing theme that the review found related to neighborhoods, specifically addressing their attractiveness, social networks, and external reputation (1). Neighborhood attractiveness and aesthetics were perceived as crucial for cognitive and mental well-being of residents (1). This is consistent with Ige-Elegbede et al. (15) systematic review findings which found that the design of the neighborhood environment was associated with health and well-being of residents. One way to improve neighborhood attractiveness could be through investing in greenspace which may contribute to improvements in both physical and mental health, such as improvements in respiratory health (16) and lower rates of depression (17).

Furthermore, the review found social support and networks within neighborhoods improved mental health in residents (1). This is consistent with Pérez et al. (18) who highlighted two systematic reviews which demonstrated a positive association between neighborhood social interaction and mental and physical health outcomes. However, Samuel et al. (19) found inconsistent evidence of a positive association between social cohesion and health. One way to engage the community and enhance social cohesion could be through engaging in co-production methods, whereby professionals and communities collaborate to develop tailored solutions for services and communities (20). The adoption of co-production approaches may improve trust and cohesion between public services and communities, hopefully leading to better health outcomes (21).

The review highlighted those residents who perceived a better internal reputation within their neighborhood had better mental health outcomes (1). This is supported by Tran et al. (22) who found residents who perceived their neighborhood to have poor reputation also had greater association with severe psychological distress. To enhance the neighborhood’s reputation; communities could promote cultural events within neighborhoods through policy initiatives which could enrich the residents' quality of life. This could be achieved by fostering a stronger sense of place, enhancing local identity, and promoting social cohesion through increased opportunities for social interaction and engagement (23–24). Moreover, such policies could contribute to the attractiveness and revitalization of neighborhoods by showcasing the diversity and creativity of the area (24–25). Urban regeneration plans could also play a role in changing the internal reputation of deprived areas, but there remains little clarity about how to measure the effects on socioeconomic and health outcomes (26–27).

#### 4.2.2 Building

The review also found outcomes related to building conditions, focusing on housing condition, temperature, density, and materials (1). Specifically, it found that poor housing conditions, including overall household disrepair, dampness, and mold resulted in poor psychological wellbeing and increased risk of respiratory problems (1). The World Health Organization (5) reports similar findings and views poor housing conditions as a critical public health priority. Wimalasena et al. (28) also supports these findings and states that the indoor housing environment has a significant impact on resident respiratory health. Furthermore, a positive association between housing refurbishment, modifications, heating, improvements to ventilation and water supply on the one hand, and respiratory outcomes, quality of life and mental health on the other has been found in the literature (29). One way housing conditions could be improved is through using local Government authority housing enforcement and selective licensing measures (30–31). In the United Kingdom, housing enforcement officers conduct inspections on privately rented properties, focusing on addressing any hazards to enhance housing safety and health using the housing health and safety rating system (HHSRS) (32). Additionally, local Government authorities could implement selective licensing schemes which aim to improve housing conditions in the private rented sector. Selective licensing is introduced in targeted areas and requires landlords to pay a license fee, and permit inspections to approve housing quality (33). Selective licensing has been found to improve area-based mental health and reduce anti-social behavior (31).

Another approach to enhance housing conditions involves Government grants. One scheme recently introduced in the United Kingdom is the Cozy Homes in Lancashire initiative (34). This scheme offers a range of grants to households to improve insulation and enhance carbon renewable technologies (34). For example, the Green Home Grant provides funding to upgrade windows, doors, insulation, and renewable technologies (34). This could enhance energy efficiency in households leading to small but significant improvements in residents’ health, particularly for those on lower incomes (35).

#### 4.2.3 Policy and Market

The review uncovered positive outcomes linked to homeownership (1). However, Chen et al. (36) found inconclusive evidence of homeownership assistance and health outcomes. Policies aimed at supporting first-time buyers may yield additional benefits. By bolstering housing affordability, these initiatives have the potential to enhance mental well-being (37). Conversely, a markedly different policy approach could involve transitioning towards a housing system akin to Germany's, where the state holds a larger share of housing, and individuals are comfortable with renting (38). As the review by Alidoust et al. (1) identified the effects of homeownership on health, it is important to note that the growth in mass homeownerships seems to have stalled around Europe, particularly for young adults who are affected by the deterioration of labor conditions accelerated by the 2007 global financial crisis (39).

### 4.3 Recommendations for future research

Given the limitations we underlined on the systematic review by Alidoust et al. (1), better focused systematic reviews, featuring a more clearly defined search strategy, a wider set of databases and more systematic screening and critical appraisal procedures are recommended in this domain of research. At the same time, given the breadth of the literature of housing impacts on health, it appears sensible to develop separate reviews (or sub-reviews) both on quantitative and qualitative evidence and on different populations. Secondly, 85% of studies in this review were quantitative suggesting a lack of included qualitative studies. This may be reflective of the topic area; however, one recommendation would be to use qualitative research methodologies to explore and synthesize people’s lived experience of housing. This would provide an additional perspective to existing research, such as highlighting important outcomes that may have been overlooked, and providing deeper explanation to findings (40). Nonetheless, the comprehensive scope of this review can be instrumental in informing predictive models assessing the impact of housing and neighborhood factors on health. This, in turn, can aid in the design of improved living environments in the future. It should be noted that we draw our recommendations for policy and future research on the basis of our RaCES critical appraisal of one systematic review. Inevitably, the scope of our recommendations will be determined by the scope of the review and the quality of the findings by Alidoust et al. (1). As such, we recognize that a more systematic process of finding evidence-based recommendations (e.g. umbrella reviews) could represent an avenue for future research.

### 4.4 Conclusion

This commentary critically appraised the systematic review by Alidoust et al. (1) on the multiple impacts of housing on health. We identified limitations with the methods used in the systematic review regarding, the use of databases, and the rigor and transparency of the processes related to screening, data extraction and quality assessment, which all indicate the need for further research on this area. We suggested implications for policy based on the review’s findings, context of current policy, and other supportive literature. In particular, we highlight a range of evidence-based initiatives aiming to improve (i) neighborhood attractiveness, social networks, and internal reputation, (ii) building conditions, temperature, density, and materials (iii) housing affordability and tenancy conditions, and (iv) labor market attractiveness which all have the potential of enhancing physical and mental health.

## Figures and Tables

**Table 1:** Critical appraisal using the Joanna Briggs Institute critical appraisal checklist for systematic reviews and research syntheses

JBI critical appraisal checklist items	Responses
1. Is the review question clearly and explicitly stated?	UNCLEAR
2. Were the inclusion criteria appropriate for the review question?	NO
3. Was the search strategy appropriate?	YES
4. Were the sources and resources used to search for studies adequate?	UNCLEAR
5. Were the criteria for appraising studies appropriate?	NO
6. Was critical appraisal conducted by two or more reviewers independently?	NO
7. Were there methods to minimize errors in data extraction?	NO
8. Were the methods used to combine studies appropriate?	UNCLEAR
9. Was the likelihood of publication bias assessed?	NO
10. Were recommendations for policy and/or practice supported by the reported data?	NO
11. Were the specific directives for new research appropriate?	YES

JBI: Joanna Briggs Institute
